# Corticosterone Contributes to Diet-Induced Reprogramming of Post-Metamorphic Behavior in Spadefoot Toads

**DOI:** 10.1093/iob/obae012

**Published:** 2024-04-24

**Authors:** A M Shephard, S R Lagon, S Jacobsen, K Millar, C C Ledón-Rettig

**Affiliations:** Department of Biology, Indiana University Bloomington, Myers Hall, 915 East 3rd Street, Bloomington IN 47405, USA; Department of Biology, Indiana University Bloomington, Myers Hall, 915 East 3rd Street, Bloomington IN 47405, USA; Department of Biology, Indiana University Bloomington, Myers Hall, 915 East 3rd Street, Bloomington IN 47405, USA; Department of Biology, Indiana University Bloomington, Myers Hall, 915 East 3rd Street, Bloomington IN 47405, USA; Department of Biology, Indiana University Bloomington, Myers Hall, 915 East 3rd Street, Bloomington IN 47405, USA

## Abstract

Stressful experiences in early life can have phenotypic effects that persist into, or manifest during, adulthood. In vertebrates, such carryover effects can be driven by stress-induced secretion of glucocorticoid hormones, such as corticosterone, which can lead to developmental reprogramming of hypothalamic-pituitary-adrenal/interrenal axis activity and behavior. Nutritional stress in the form of early life nutrient restriction is well known to modify later life behaviors and stress activity through corticosterone-related mechanisms. However, it is not known whether corticosterone is also mechanistically involved in carryover effects induced by a different form of nutritional variation: the use of alternate or entirely novel types of dietary resources. The plains spadefoot (*Spea bombifrons*) presents an excellent system for testing this question, since larvae of this species have evolved to use 2 alternate diet types: an ancestral detritus-based diet and a more novel diet of live shrimp. While previous work has shown that feeding on the novel shrimp diet influences juvenile (i.e., post-metamorphic) behavior and corticosterone levels, it is unclear whether these diet-induced carryover effects are mediated by diet-induced corticosterone itself. To test for the mechanistic role of corticosterone in diet-induced carryover effects, we experimentally treated *S. bombifrons* larvae with exogenous corticosterone and measured subsequent effects on juvenile behavior and corticosterone levels. We found that while shrimp-fed larvae had elevated corticosterone levels, treatment of larvae with corticosterone itself had effects on juvenile behavior that partially resembled those carryover effects induced by the shrimp diet, such as altered food seeking and higher locomotor activity. However, unlike carryover effects caused by the shrimp diet, larval corticosterone exposure did not affect juvenile corticosterone levels. Overall, our study shows that corticosterone-related mechanisms are likely involved in carryover effects induced by a novel diet, yet such diet-induced carryover effects are not driven by corticosterone alone.

## Introduction

Stressful environments early in life can have lasting effects on the development of adult phenotypes. Such stress-induced carryover effects have been shown to influence a range of traits (e.g., physiology, morphology, and behavior) and can have major fitness consequences (Monaghan 2008; Bateson et al. 2014; Monaghan and Haussmann 2015). However, to fully understand the developmental and evolutionary implications of these carryover effects, it is critical to understand the physiological mechanisms linking early life stress to adult phenotypic outcomes (O'Connor et al. 2014).

One type of physiological mechanism that can underlie carryover effects of stressful early life environments on adult phenotypes is hormonal pathways (Spencer et al. 2009). A widely studied example of such a hormonal mechanism is the vertebrate hypothalamic-pituitary-adrenal/interrenal (HPA/HPI) axis (Romero 2004). A key component of the HPA/HPI axis involves the elevated secretion of glucocorticoid hormones, such as corticosterone, under challenging or stressful conditions (Romero and Butler 2007; Denver 2009a). Elevated corticosterone during early life can result in developmental “reprogramming” of the HPA/HPI axis, leading to altered stress response activity, metabolic changes, and behaviors later in life (Seckl 2001; Seckl and Meaney 2004). Such corticosterone-induced carryover effects have been documented in response to a wide breadth of environmental conditions, such as predator presence (Middlemis Maher et al. 2013; Harris and Carr 2016), cues from the social environment (Anisman et al. 1998; Nishi et al. 2014), and resource limitation (Hu et al. 2008; Schmidt et al. 2014).

The role of corticosterone in mediating carryover effects induced by early life nutrient restriction has been particularly well-studied (MacDougall-Shackleton and Spencer 2012; Crino and Breuner 2015; Wada and Coutts 2021). For instance, periods of nutrient restriction during development can increase corticosterone levels in amphibians (Crespi and Denver 2005), birds (Corbel and Groscolas 2008; Honarmand et al. 2010; Sprague and Breuner 2010), and mammals (Cuervo et al. 2016). Specific carryover effects associated with early life nutrient restriction include increased activity levels, food-seeking behaviors, and modified corticosterone activity in adulthood (Breier et al. 2001; Hu et al. 2008; Roth et al. 2012). Despite this previous work on nutrient restriction, much less is known about the mechanistic involvement of corticosterone in mediating carryover effects induced by another type of nutritional variation: the consumption of alternate or even novel dietary resource types. In nature, exposure to alternate dietary resources occurs in generalist feeders or during dietary niche shifts, where populations begin to feed on novel sources of nutrition (Snell-Rood et al. 2018; Sikkink et al. 2020; Leonard and Lancaster 2022).

In this study, we asked whether larval corticosterone is involved in carryover effects induced by the use of a novel diet during development. We experimentally tested this hypothesis using the plains spadefoot (*Spea bombifrons*), whose larvae exhibit a resource polyphenism. Specifically, *S. bombifrons* larvae develop as either an omnivore morph that feeds on an ancestral detritus-based diet or a carnivore morph that feeds on a live shrimp diet (Pomeroy 1981). In nature, spadefoot larvae develop in ephemeral ponds, and the resource polyphenism reduces competition among larvae and increases the likelihood that they will grow large enough to metamorphose before the ponds dry (Pfennig 1990, 1992). The frequency at which larvae develop the polyphenism is influenced by pond characteristics such as shrimp density, ephemerality, and shallowness (Pfennig 1990), as well as the degree of resource competition with heterospecifics (Pfennig et al. 2006). While shrimp-feeding carnivores are better able to survive in ponds that dry faster (Pfennig 1992), feeding on the shrimp diet has been shown to induce post-metamorphic carryover effects that are putatively maladaptive, such as delayed movement in a novel environment and poorer prey-catching abilities (Ledón-Rettig and Lagon 2021). Additionally, larvae developing on the shrimp-based diet develop into juveniles with higher corticosterone levels (Ledón-Rettig and Lagon 2021) and altered HPI axis regulatory responses to early life nutrient restriction (Shephard et al. 2024). Diet-induced carryover effects on corticosterone physiology are still present 1 year following metamorphosis (Ledón-Rettig et al. 2023). While these observations suggest a mechanistic role for corticosterone in larval diet–induced carryover effects, the extent to which such effects are mediated by larval diet–induced corticosterone is unclear.

To test whether corticosterone is mechanistically involved in diet-induced carryover effects on post-metamorphic (i.e., juvenile) behavior and glucocorticoid regulation in *S. bombifrons*, we used a “phenotypic engineering” approach (Ketterson et al. 1996). Specifically, we manipulated larval corticosterone levels and tested the extent to which this manipulation recapitulates previously observed carryover effects of the shrimp diet (Ledón-Rettig and Lagon 2021). First, to test whether feeding on the novel shrimp diet increases larval corticosterone, we compared corticosterone levels across larvae reared on either shrimp or the ancestral diet of detritus. Second, to validate our phenotypic engineering approach, we exposed larvae to exogenous corticosterone and subsequently measured endogenous larval corticosterone levels. Third, we tested whether treatment of larvae with corticosterone elicits carryover effects on juvenile behavior and glucocorticoid regulation that recapitulate effects induced by the shrimp-based diet (Ledón-Rettig and Lagon 2021). With respect to behaviors, we specifically measured the effects of larval corticosterone on juvenile behaviors related to food seeking, prey capture ability, boldness, exploration, and activity levels. Given significant interest in individual repeatability of behavioral traits (Bell et al. 2009), we tested for within-individual repeatability of behavioral carryover effects by quantifying juvenile behavior in two separate trials across juvenile development. Consistent with previous carryover effects observed in juvenile *S. bombifrons* that developed on the shrimp-based diet (Ledón-Rettig and Lagon 2021), we expected that corticosterone-treated individuals would display poorer prey capture abilities and lower boldness, yet higher locomotor activity and exploration. Additionally, we measured carryover effects of larval corticosterone exposure on juvenile stress reactivity, with the expectation that individuals exposed to corticosterone as larvae would display modified corticosterone regulation at the juvenile stage.

## Methods

### Experimental overview and origin of study animals

Using *S. bombifrons*, we combined three experiments to test the mechanistic role of corticosterone in mediating carryover effects induced by feeding on a novel shrimp-based diet. In Experiment 1, we tested whether the shrimp diet increases endogenous corticosterone levels of larvae. In Experiment 2, we tested whether treating larvae with corticosterone itself increases endogenous larval corticosterone levels. In Experiment 3, we tested for carryover effects of larval corticosterone treatment on behavior and stress reactivity of juvenile frogs.

All larvae used in these experiments originated from adult frogs collected from Willcox, AZ, in 2018. These adult frogs have been maintained in a colony at Indiana University Bloomington and housed in a temperature-controlled room at approximately 25.7°C on a 12-h light/dark photoperiod. All frogs were housed in this room for breeding and subsequent experimentation with larvae and juveniles described below. All animal husbandry and experimental procedures were approved by the Indiana University Institutional Animal Care and Use Committee (IACUC protocol #21–011).

### Breeding protocols and microcosm setup

To produce larvae for each experiment, we injected mating pairs with luteinizing hormone–releasing hormone (LHRH; GenScript RP11937). Females were injected with 40–100 µL of 1 µg/100 µL LHRH, and males were injected with 20–50 µL of 1 µg/100 µL LHRH. We placed mating pairs in individual plastic containers with filtered, dechlorinated water that was allowed to sit overnight (hereafter “aged water”). We allowed mating pairs to breed overnight, and we allowed eggs to incubate for 48 h until hatching. We reared the resulting larvae in plastic microcosms (18 × 11 × 14 cm) containing ∼800 mL of aged water.

### Experiment 1: Effects of the novel diet on larval endogenous corticosterone levels

We first conducted an experiment to test whether consuming a novel shrimp-based diet increases corticosterone levels of spadefoot larvae. In August 2022, larvae from a single breeding pair were randomly assigned to either a detritus- or shrimp-based diet immediately upon transfer to rearing microcosms. The detritus treatment consisted of finely ground cichlid feed (25% protein, 4% fat, 5% fiber; Hikari, Hayward, California, USA), which resembles the nutritional composition of the detrital diet *S. bombifrons* consumes in the wild (Pfennig et al. 1991). The shrimp treatment consisted of live brine shrimp (*Artemia*), which resembles the shrimp fed on by *S. bombifrons* in the wild (Pomeroy 1981). Within each diet treatment, we reared larvae at either a high density (eight larvae per microcosm) or a low density (three larvae per microcosm). Density treatments were determined based on preliminary observations of tadpole densities that do not significantly decrease microcosm water quality. We note that this density treatment was not a central focus of our study and that the density manipulation was included because these data were repurposed from a different unpublished dataset. We provided larvae in all microcosms with *ad libitum* food. Each diet-by-density treatment combination contained 15 replicate microcosms. Final larval sample sizes for this analysis were as follows: shrimp-fed high density (*n* = 30 larvae), shrimp-fed low density (*n* = 30 larvae), detritus-fed high density (*n* = 31 larvae), and detritus-fed low density (*n* = 26 larvae).

Twelve or 13 days after hatching, we performed a waterborne hormone assay to test the effects of dietary treatment on larval endogenous corticosterone levels. This assay has shown that waterborne corticosterone levels correlate positively with plasma corticosterone levels in juvenile *S. bombifrons* (Ledón-Rettig et al. 2023). We evenly distributed the two dietary treatments across the 2 days of assays. We selected two larvae from each microcosm: the largest larva and the second largest larva. Prior to the start of the assay, we removed individual larvae from their rearing microcosm and briefly rinsed them in aged water before transferring them to a plastic cup filled with 40 mL of aged water for a period of 60 min. Upon completion of the assay, we removed larvae and stored water samples at –20°C until further processing (see the “Corticosterone extractions and enzyme-linked immunosorbent assay” section).

### Experiment 2: Effects of exogenous corticosterone on endogenous larval corticosterone levels

In a parallel experiment using *S. bombifrons* larvae from the same clutch as Experiment 1, we tested whether treatment of spadefoot larvae with exogenous corticosterone increases endogenous larval corticosterone levels. Here, we reared larvae singly in microcosms with *ad libitum* access to both detritus and live shrimp. Seven days after transferring larvae to rearing microcosms, we treated the 800 mL microcosms with one of three treatments (*n* = 30 larvae per treatment): (1) an exogenous corticosterone treatment (200 μL of 1 mM corticosterone [Cayman Chemical 16063] dissolved in ethanol [EtOH], for a final concentration of 250 nM corticosterone), (2) a control treatment of 200 μL EtOH, or (3) a control treatment of 200 µL water. The dosage for the corticosterone treatment was chosen based on previous studies in spadefoot larvae (Ledón-Rettig et al. 2009, 2010). We repeated treatments daily for 7 consecutive days, which corresponded to ∼20% of the development time of tadpoles in our study population. Upon completion of the hormonal treatment, larvae were subjected to the waterborne hormone assay to measure endogenous corticosterone levels as described in Experiment 1. Following completion of the waterborne hormone assay, water samples were immediately stored at –20°C until corticosterone extractions (see the “Corticosterone extractions and enzyme immunosorbent assay” section).

### Experiment 3: Carryover effects of larval corticosterone on juvenile behavior and corticosterone regulation

In the third experiment, we tested for carryover effects of larval corticosterone treatment on behavior and corticosterone regulation in juvenile spadefoots. We reared larvae from clutches derived from an additional mating pair in October 2022. We transferred newly hatched larvae (48 h post-mating) to microcosms with 800 mL of aged water and with *ad libitum* access to both detritus and shrimp. Seven days later, we assigned larvae to either an exogenous corticosterone treatment or EtOH control treatment for 7 consecutive days, as described in Experiment 2. To confirm that we successfully modified corticosterone levels with our corticosterone treatment, we used a waterborne assay on all larvae 1–2 days after discontinuing treatments; the treatments were randomly distributed over the 2 days of assays. Following these assays, we retained larvae in their individual microcosms for the duration of their larval growth period until the onset of metamorphosis.

At the onset of metamorphosis (i.e., the date of forearm emergence), we transferred larvae to new microcosms that contained sand on one side and ∼1 cm of water on the other side. Upon completion of metamorphosis (i.e., the date of tail resorption), each microcosm was evenly covered with ∼2 cm of moist sand, and juveniles were fed live crickets (Josh's Frogs; Owosso, MI). For each juvenile, we measured both weight and snout–vent length (SVL) at metamorphosis. We fed juveniles live crickets supplemented with Repti Calcium (Zoo Med) and Herptivite (Rep-Cal) and exchanged their sand with new sand approximately 5 weeks post-metamorphosis. We kept these juveniles in their individual microcosms until the behavioral and hormonal assays described below.

Three to 4 weeks after metamorphosis, we assayed all juveniles for carryover effects on behavioral traits (larval EtOH treatment: *n* = 20 juveniles; larval corticosterone treatment: *n* = 19 juveniles). Nine to 10 weeks after metamorphosis, we repeated the behavioral assays to determine the repeatability of any behavioral carryover effects. All behavioral assays were conducted in plastic arenas (29 cm length × 19 cm width × 17.5 cm height) containing ∼7.5 cm of moist sand, and all assays were conducted in a behavioral room maintained at ∼25°C. A researcher (S.R.L for the first set of recordings, C.L-R. for the second) placed each juvenile in its own arena, added 10 small crickets, and then quickly left the room. After we recorded juveniles’ behaviors with a digital video camera for 10 min, we returned juveniles to their home microcosm. We then sacrificed juveniles and collected their fat bodies for imaging analysis.

We used both human observation and software to quantify juvenile behaviors. First, a researcher who was blind to the juveniles’ treatments (S.R.L. for the first set of recordings, S.W.J. for the second) measured the following: latency to move (the time of an initial movement after the start of the assay), total prey strikes (total number of successful and unsuccessful strikes at crickets displayed by a juvenile over the assay), and prey strike efficiency (successful prey strikes divided by total prey strikes). Second, we used the software ToxTrac (Rodriguez et al. 2018) to measure each juvenile's exploration rate (the number of areas of the arena visited by a juvenile), distance traveled, average speed, and average acceleration.

Five to 6 weeks after metamorphosis, between behavioral trials, we used juveniles from our carryover experiment (Experiment 3) in a waterborne hormone assay that captured corticosterone levels at two time points: pre-stress induction (i.e., baseline corticosterone levels) and post-stress induction (i.e., stress-induced corticosterone levels). We captured baseline corticosterone levels by placing juveniles singly in small enclosures (12 cm length × 6.5 cm width × 6.5 cm height) filled with 50 mL of aged water and allowing them to soak undisturbed for 30 min, after which water was removed and immediately stored at –20°C. We then refreshed enclosures with another 50 mL of water and subjected individuals to a stress treatment consisting of eight cycles of gentle rocking (1 min) and rest (3 min), for a total of 32 min. Following this stress treatment, we refreshed water in each enclosure and allowed individuals to rest for 30 min. Following this initial rest period, we refreshed water in each enclosure again before a final rest period of 30 min. We stored water samples from this final rest period at –20°C and used them for our measures of stress-induced corticosterone levels. Final juvenile sample sizes for corticosterone analyses were as follows: EtOH treatment (*n* = 16) and corticosterone treatment (*n* = 14).

### Corticosterone extractions and enzyme-linked immunosorbent assay

We extracted corticosterone from water samples collected from larval and juvenile *S. bombifrons*. Prior to extractions, all water samples were thawed at room temperature. We performed each corticosterone extraction from 5 mL of water sample using a vacuum manifold (Chromabond SPE, 730151B) with Sep-Pak C18 extraction columns (Waters, WAT043395), as described in Ledón-Rettig et al. (2023), but our corticosterone extracts were dried using oxygen rather than nitrogen. All extracted samples were stored at –20°C until the enzyme-linked immunosorbent assay (ELISA).

To quantify corticosterone concentrations of our extracted samples, we performed ELISA (Cayman 501320) following the manufacturer's instructions. Larval samples were first resuspended at a 40× dilution in 5% EtOH and 95% ELISA assay buffer and were then further diluted to 800× prior to plating for ELISA. Juvenile samples were resuspended in 10 µL of EtOH and 190 µL of assay buffer prior to plating. For Experiment 1, larval samples were run across four plates (average intra-assay coefficient of variation (CV) = 17.23%). For Experiment 2, larval samples were run across three plates (average intra-assay CV = 16.01%). For Experiment 3, juvenile samples were run across seven plates (average intra-assay CV = 3.31%). We validated our waterborne corticosterone assay by assessing precision, sensitivity, accuracy, and specificity of the assay (details provided in Supplementary Methods).

### Statistical analyses

We performed all statistical analyses in R Studio v. 3.6.3. For Experiment 1 (effects of diet on endogenous larval corticosterone levels), we constructed a linear mixed-effects model using the “lmer” function in the *lme4* package (Bates et al. 2015). This model tested for the fixed effect of diet (detritus or shrimp) on larval corticosterone concentration, controlling for variation in microcosm density as a discrete variable (high or low) and relative larval size as a discrete variable (i.e., whether the individual was the larger or smaller tadpole from each microcosm). We also tested for the interaction between diet and density. Microcosm ID was included as a random effect. For Experiment 2 (effects of exogenous corticosterone on endogenous larval corticosterone levels), we used an analysis of variance (ANOVA) to test the effect of hormone treatment (corticosterone, EtOH, or H_2_O control) on larval corticosterone concentration. We included larval mass as a covariate in this model. Prior to both analyses, larval corticosterone concentration data were log-transformed to improve normality. We tested for significance in each model using Type II sums of squares tests with the “Anova” function in the *car* package (Fox et al. 2012). For the model in Experiment 2, *post hoc* comparisons between hormone treatment levels were analyzed using the *emmeans* package (Lenth et al. 2019).

To test for behavioral carryover effects in Experiment 3, we first used the scaled mass index (Peig and Green 2009) to calculate juvenile body condition at metamorphosis, using juvenile body mass and SVL measurements. We then used ANOVA to test the carryover effect of larval hormone treatment on juvenile scaled mass index. Next, for each behavioral trait measured in Experiment 3 (effects of larval corticosterone on juvenile behavior and corticosterone levels), we constructed a linear mixed-effects model. These models were used to analyze the following behaviors: latency to move, total prey strikes, prey strike efficiency, distance traveled, average speed, average acceleration, and exploration rate. In each model, we included the fixed effects of larval hormone treatment (EtOH or corticosterone), behavioral trial, and juvenile mass measured at the start of each behavioral assay. Each model included individual ID as a random effect. For the model testing for effects on latency, we log-transformed latency data prior to analysis to improve normality. For the model testing for effects on total prey strikes, we used a Poisson distribution within the “glmer” function. Finally, in the strike efficiency model, we used a binomial distribution within the “glmer” function, given that strike efficiency was quantified as a proportion.

Given that some behavioral traits were correlated among individuals, we performed a principal component analysis (PCA) of latency, total prey strikes, prey strike efficiency, distance traveled, average speed, average acceleration rate, and exploration rate. We then used a linear mixed-effects model to test for the effects of larval hormone treatment on juvenile behavior PC1 score. Juvenile mass was included as a fixed effect, and behavioral trial was included as a random effect.

To assess repeatability of behaviors in Experiment 3, we used the *rptR* package (Stoffel et al. 2017) to compute intraclass correlations for behaviors of individuals sampled across the two behavioral trials. We calculated repeatability of each behavior separately for the EtOH and corticosterone treatments. Each estimate of repeatability was derived from a model including individual ID as a random effect and behavioral trial number (first or second) as a fixed effect. We used Gaussian distributions for repeatability analyses of latency behavior, exploration rate, and distance, speed, and acceleration of movement. The repeatability estimate for total strikes was analyzed using a Poisson distribution, and the estimate for strike efficiency was analyzed using a binomial distribution.

To test whether our corticosterone treatment caused physiological carryover effects, we used a linear mixed-effects model where larval corticosterone treatment, juvenile stress treatment (i.e., baseline or stress-induced), and their interaction were used as explanatory variables and juvenile waterborne corticosterone level was the response variable. In this model, we used individual ID as a random effect to control for repeated measures of baseline and stress-induced corticosterone levels within individual frogs. We also included juvenile body mass as a fixed effect. In addition to these juvenile corticosterone analyses, we tested whether larval corticosterone levels correlated with juvenile baseline or stress-induced corticosterone levels or any of the behaviors listed in Table 1. However, larval corticosterone did not significantly correlate with any of these variables (Table S1).

**Table 1:** Linear mixed-effects model results for effects of larval hormone treatment (EtOH or corticosterone) on behaviors in juvenile spadefoot toads

**Fixed effects**	**χ*^2^***	***df***	***P***
**Latency to move**			
Larval hormone treatment	1.88	1	0.17
Behavioral trial	0.00080	1	0.98
Juvenile body mass	0.50	1	0.48
**Total prey strikes**			
Larval hormone treatment	8.64	1	0.0033
Behavioral trial	1.07	1	0.30
Juvenile body mass	0.033	1	0.86
**Prey strike efficiency**			
Larval hormone treatment	0.25	1	0.61
Behavioral trial	2.82	1	0.093
Juvenile body mass	0.29	1	0.59
**Distance traveled**			
Larval hormone treatment	3.25	1	0.072
Behavioral trial	242.26	1	<0.001
Juvenile body mass	2.86	1	0.091
**Average speed**			
Larval hormone treatment	2.51	1	0.11
Behavioral trial	100.89	1	<0.001
Juvenile body mass	3.27	1	0.070
**Average acceleration**			
Larval hormone treatment	4.01	1	0.045
Behavioral trial	52.62	1	<0.001
Juvenile body mass	1.57	1	0.21
**Exploration rate**			
Larval hormone treatment	0.060	1	0.81
Behavioral trial	40.13	1	<0.001
Juvenile body mass	0.00040	1	0.98

Note: Each model includes fixed effects of behavioral trial and juvenile mass at the start of the assay, as well as the random effect of individual ID.

Since our main goal was to test whether the larval corticosterone treatment recapitulates carryover effects of the shrimp diet, we reanalyzed data from Ledón-Rettig and Lagon (2021) to determine the effects of the shrimp diet on juvenile behaviors that were not reported in that previous study, specifically food-seeking behavior (i.e., total prey strikes) and locomotor behaviors (i.e., distance, speed, and acceleration of movement). For the total prey strikes model, we used the “glmer” function as described above, including larval diet and juvenile body mass as fixed effects and observation as a random effect. To analyze effects on distance traveled, average speed, and average acceleration, we created three different linear models including the fixed effects of larval dietary treatment and juvenile body mass.

## Results

### Experiment 1: Diet effects on larval corticosterone levels

**Fig. 1:**
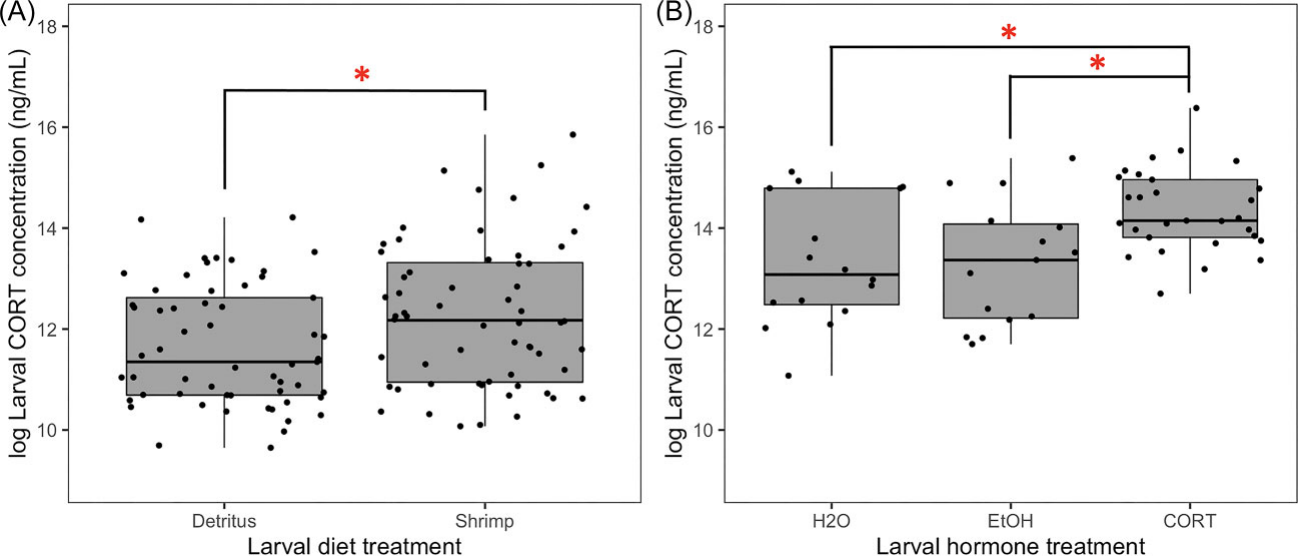
(**A**) Effect of larval diet type and (**B**) effect of larval exogenous corticosterone (CORT) exposure on CORT levels of plains spadefoot larvae (*Spea bombifrons*) as measure by a waterborne hormone assay. * indicates *P* < 0.05.

Relative to larvae developing on the detritus-based diet, larvae developing on the shrimp diet had elevated endogenous corticosterone levels (*F* = 4.60, *P* = 0.036; Fig. 1A). We found no significant effect of larval rearing density (*F* = 2.03, *P* = 0.16) on endogenous corticosterone levels of larvae. Additionally, there was no significant interaction between larval diet and rearing density on corticosterone levels (*F* = 0.92, *P* = 0.34). Larval mass did not significantly correlate with endogenous corticosterone levels (Spearman's rho = 0.26, *P* = 0.11).

### Experiment 2: Effects of hormone treatment on larval corticosterone levels

Overall, we found a significant effect of larval hormone treatment on endogenous larval corticosterone levels (*F* = 3.78, *P* = 0.03). *Post hoc* comparisons showed that larvae treated with corticosterone displayed higher endogenous corticosterone levels than both EtOH-treated larvae (Estimate = 1.69, *t* = 2.47, *P* = 0.043; Fig. 1B) and H_2_O-treated larvae (Estimate = 1.48, *t* = 2.70, *P* = 0.024). H_2_O- and EtOH-treated larvae did not differ in terms of endogenous corticosterone levels (Estimate = −0.22, *t* = −0.53, *P* = 0.86; Fig. 1B), indicating that the EtOH vehicle itself did not affect larval corticosterone levels.

### Experiment 3: Carryover effects of larval hormone treatment on juvenile behavior and corticosterone levels

**Fig. 2:**
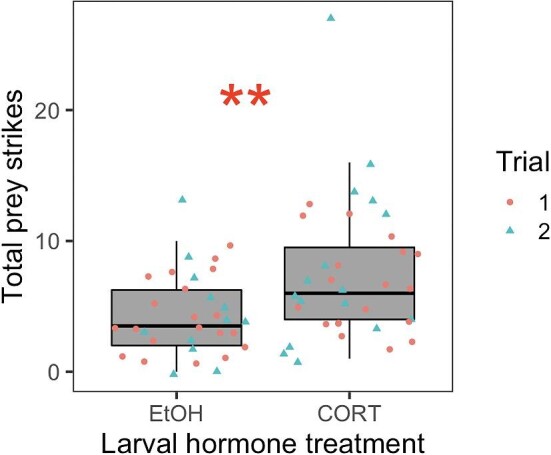
Carryover effect of larval corticosterone exposure on total prey strikes (a measure of food-seeking behavior) exhibited by juvenile plains spadefoots (*Spea bombifrons*). Prey strikes were quantified as the number of times a juvenile frog struck at a cricket prey item, either successfully or unsuccessfully, during a 10-min behavioral trial. Total prey strikes (a measure of food-seeking behavior) indicate the number of times an individual struck at a cricket, either successfully or unsuccessfully. Each point corresponds to the score of an individual frog's behavior in one of two separate trials occurring 3–4 weeks after metamorphosis and 9–10 weeks after metamorphosis. ** indicates *P* < 0.01.

**Fig. 3:**
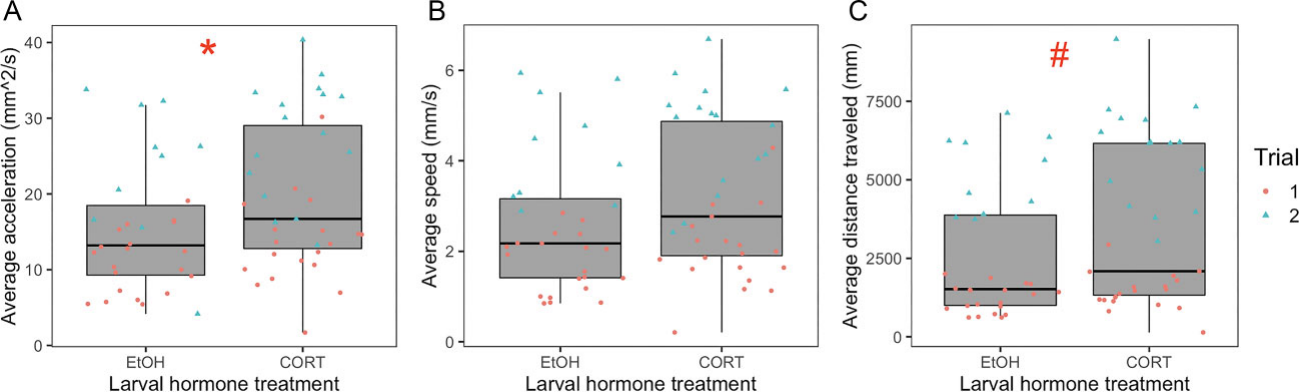
Carryover effect of larval hormone treatment on three measures of locomotor activity in juvenile plains spadefoots (*Spea bombifrons*): average acceleration (**A**), average speed (**B**), and distance traveled (**C**). To measure locomotor activity, juveniles were placed individually in arenas with 10 small crickets (prey items) for 10 min. Measures of activity were quantified using ToxTrac software (Rodriguez et al. 2018). Each point corresponds to the score of an individual frog's behavior in one of two separate trials occurring 3–4 weeks after metamorphosis and 9–10 weeks after metamorphosis. * indicates *P* < 0.05. # indicates 0.05 < *P* < 0.1.

We tested for carryover effects of larval corticosterone treatment on juvenile behaviors related to boldness (latency to move), food seeking (total number of prey strikes), prey capture ability (prey strike efficiency), exploration rate, and locomotor activity (distance traveled, average speed, and average acceleration). Juveniles that were exposed to corticosterone at the larval stage displayed a higher total number of prey strikes (Fig. 2), but prey strike efficiency was not affected by larval corticosterone treatment (Table 1). Additionally, juveniles treated with corticosterone as larvae displayed greater average acceleration (Fig. 3A) and tended to travel further in behavioral assays (Fig. 3B and C). We found no effects of larval hormone treatment on juvenile latency to move or exploration rate (Table 1). Additionally, there was no effect of larval hormone treatment on juvenile body condition (i.e., the scaled mass index; *F* = 0.036, *P* = 0.85) or fat body area (*F* = 1.06, *P* = 0.31).

Our PCA showed that PC1 explained 48.63% of the behavioral variation among individuals, and higher PC1 scores corresponded with longer latency to move, higher strike efficiency, and greater exploration rate, but fewer prey strikes and reduced distance, speed, and acceleration (Fig. S1A). Relative to EtOH-treated individuals, we found that juveniles exposed to the larval corticosterone treatment displayed significantly lower juvenile behavioral PC1 scores (Fig. S1B; χ^2^ = 5.51, *P* = 0.019), indicating shorter latency and reduced prey strike efficiency and exploration rate, but higher prey strikes and distance, speed, and acceleration of movement.

For most repeatability estimates of juvenile behavioral traits, 95% confidence intervals overlapped with zero (Table S2), suggesting that most behavioral responses were not significantly repeatable across trials. However, we did find that that latency to move was significantly repeatable among individuals that were treated with EtOH as larvae. On average, juveniles were ∼7% heavier at Behavioral Trial 2 (mean mass = 1.13 g, SD = 0.23) than at Behavioral Trial 1 (mean mass = 1.05 g, SD = 0.17). There was no significant interaction between larval treatment and behavioral trial on juvenile body mass (χ^2^ = 0.92, *P* = 0.34).

**Fig. 4:**
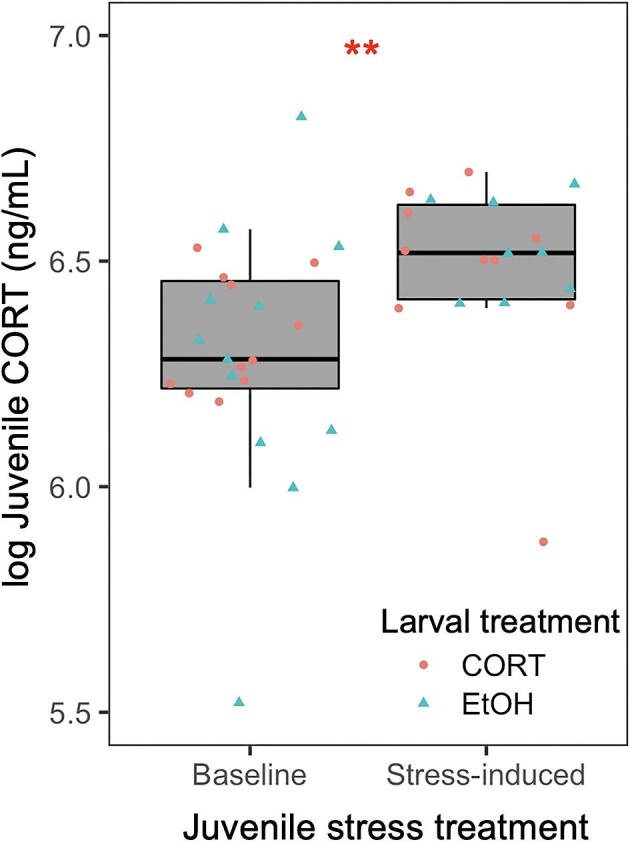
Effect of juvenile stress treatment (baseline or stress-induced) on waterborne corticosterone (CORT) levels of juvenile plains spadefoots (*Spea bombifrons*). All individuals underwent a larval treatment of either EtOH exposure or exogenous CORT exposure. ** indicates *P* < 0.01. Waterborne CORT values are not adjusted for mass because there was no correlation between CORT and juvenile mass (Spearman's rho = −0.11, *P* = 0.50).

We found no significant effect of exogenous larval corticosterone exposure on juvenile waterborne corticosterone levels (Table 2). However, we did find that the juvenile stress treatment was sufficient to increase juvenile corticosterone (Table 2 and Fig. 4). There was no significant interaction between larval corticosterone exposure and juvenile stress treatment on corticosterone levels (Table 2). Additionally, we found no significant correlation between juvenile body mass and corticosterone (Spearman's rho = −0.11, *P* = 0.50).

**Table 2:** Linear mixed-effects model results for effects of larval hormone treatment (EtOH or corticosterone) and juvenile stress treatment (baseline or stress-induced) on plains spadefoot (*Spea bombifrons*) juvenile corticosterone levels, controlling for variation in juvenile body mass

**Fixed effects**	**χ^2^**	***df***	***P***
Larval hormone treatment	0.049	1	0.82
Juvenile stress treatment	8.52	1	0.0035
Larval hormone treatment × Juvenile stress treatment	0.59	1	0.44
Juvenile body mass	0.39	1	0.53

Our reanalysis of data from Ledón-Rettig and Lagon (2021) showed that individuals reared on the shrimp diet had greater acceleration of movement as juveniles than individuals reared on the detritus diet (Fig. S2). However, larval diet type did not affect juvenile distance traveled, average speed, or total prey strikes (Table S3).

## Discussion

**Fig. 5:**
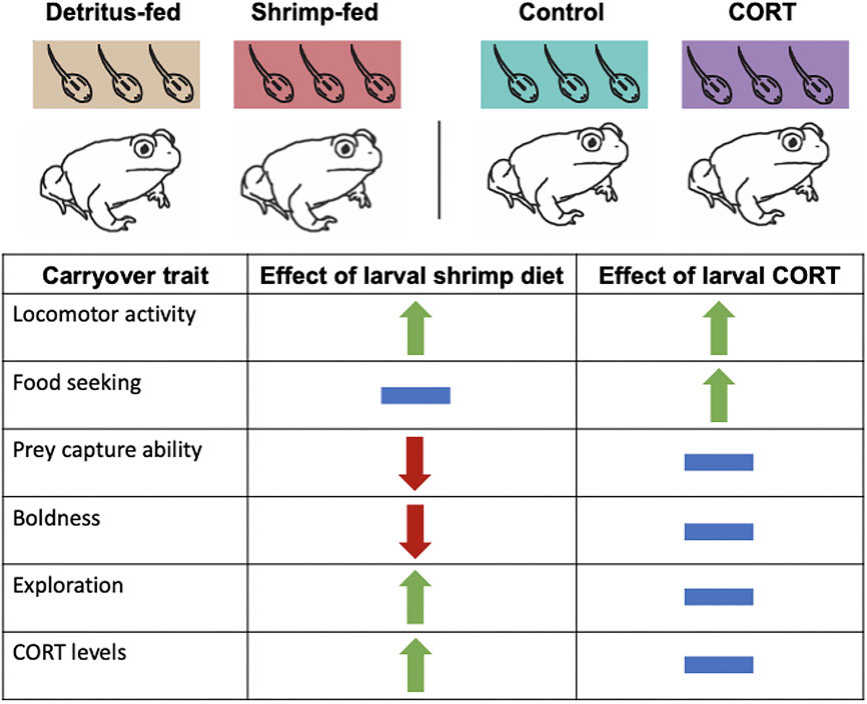
Carryover effects of larval corticosterone (CORT) exposure observed in the current study are compared with results from a previous study by Ledón-Rettig and Lagon (2021) testing for carryover effects of a larval shrimp diet relative to the effects a detritus diet. Arrows pointing up indicate that the larval treatment had a positive effect on the carryover trait. Arrows pointing down indicate a negative effect on the carryover trait. Dashes indicate that the treatment had no effect on the carryover trait.

Nutritional stress in the form of early life nutrient restriction is known to alter later life behavior and HPA/HPI axis activity through glucocorticoid mechanisms (Hu et al. 2008; MacDougall-Shackleton and Spencer 2012; Crino and Breuner 2015; Wada and Coutts 2021). However, less is known about whether glucocorticoid mechanisms are also involved in carryover effects induced by the use of alternate or novel diet types. Here, we addressed this question using the plains spadefoot (*S. bombifrons*), a species capable of consuming either an ancestral diet of detritus or a more novel diet of live shrimp (Pomeroy 1981). Specifically, we tested whether carryover effects of larval corticosterone exposure on juvenile behavior and stress reactivity recapitulate effects previously shown to be induced by the novel shrimp diet (Ledón-Rettig and Lagon 2021). We found that, relative to detritus-fed larvae, shrimp-fed larvae indeed had elevated corticosterone levels (Fig. 1A), consistent with previous hypotheses (Ledón-Rettig et al. 2010; Levis et al. 2022). Additionally, in larvae reared on a diet of mixed detritus and shrimp, we found that exposure of larvae to corticosterone itself was sufficient to elevate larval corticosterone levels (Fig. 1B). Yet, treatment of larvae with exogenous corticosterone itself affected juvenile behaviors (Figs. 2 and 3) in ways that only partly resemble those effects of the shrimp diet (Fig. 5). Further, we found no effect of larval corticosterone exposure on juvenile corticosterone (Table 2).

Our results suggest that at least one behavioral carryover effect is mediated by diet-induced corticosterone (Fig. 5). Specifically, we found that individuals treated with corticosterone as larvae developed into juveniles that displayed increased locomotor activity in terms of distance and acceleration of movement (Fig. 3). This matches patterns of locomotor behavior observed in shrimp-reared juveniles, which displayed greater acceleration of movement than detritus-fed individuals (Fig. S2). Despite these similarities in behavior across studies, larval corticosterone treatment did not recapitulate other behavioral carryover effects induced by the shrimp diet, including boldness (measured as latency to move), prey capture ability (measured as prey strike efficiency), and exploratory behavior, when those behaviors were assessed individually (Fig. 5). However, our PCA indicated that the larval corticosterone treatment significantly reduced juvenile PC1 score, corresponding to shorter latency, reduced prey striking ability, and lower exploration rate, but also increased total prey strikes as well as distance, speed, and acceleration of movement (Fig. S1). This result is consistent with the idea that the larval corticosterone treatment induced a post-metamorphic behavioral syndrome.

Our finding that larval corticosterone exposure only partly recapitulates the behavioral effects of the shrimp diet suggests that mechanisms independent of corticosterone likely also contribute to diet-induced carryover effects. For example, the nutritional composition of the shrimp diet might influence behavior through effects of dietary protein or lipid intake on brain development. Indeed, dietary protein levels can directly influence behavior in both vertebrates (DeNapoli et al. 2000; Van Emous et al. 2014) and invertebrates (Bouchebti et al. 2022), and nutritional variation in general has long been recognized as an evolutionary driver of brain evolution across species (see Snell-Rood et al. 2020 for references). Additionally, it is possible that engagement in behaviors necessary to consume a live shrimp diet at the larval stage could be another corticosterone-independent mechanism influencing juvenile behaviors. For instance, feeding on diets that are difficult to locate or obtain has been implicated as a selective driver of neural investment (Clutton-Brock and Harvey 1980; Farris and Schulmeister 2011), and engagement in foraging activities has been shown to influence brain development in a variety of taxa (Rehan et al. 2015; Oldfield et al. 2020). Finally, it is possible that hormones other than corticosterone could contribute to diet-induced carryover effects. For example, thyroid hormone is produced in larval amphibians in response to environmental stress and can influence timing of metamorphosis and other performance-related traits (Denver 2009b).

Although corticosterone-treated larvae developed into juveniles that displayed some behaviors consistent with shrimp-reared individuals, we also found that larval corticosterone exposure induced behavioral effects beyond those previously shown to be induced by the shrimp diet. For example, we found that juveniles exposed to larval corticosterone displayed greater food-seeking behavior in terms of total number of prey strikes (Fig. 2)—a carryover effect not shown to be induced by the shrimp diet (Fig. 5; Ledón-Rettig and Lagon 2021). This effect of larval corticosterone on juvenile prey striking behavior resembles hyperphagia (i.e., increased hunger or food seeking), which is a more general response to early life nutrient stress and HPA/HPI axis reprogramming in vertebrates (Bruce et al. 1982; Tokuyama and Himms-Hagen 1987; Koch et al. 2002; Orozco-Solís et al. 2011).

Despite elevated food-seeking behavior in corticosterone-treated individuals, we did not find an effect of corticosterone treatment on juvenile fat levels or body mass at the time of either behavioral assay. One potential explanation for the lack of observed effects of larval corticosterone on juvenile fat and body mass could be that corticosterone-treated individuals were less effective at building or maintaining energy reserves, as they tended to be more active overall (Fig. 3), yet did not show increases in prey capture ability (Fig. 5). We note that the feeding ecology of *S. bombifrons* juveniles, which consists of capturing small, fast-moving prey items (e.g., insects), might make food acquisition more difficult than in other contexts where hyperphagic responses to early life stress have been studied, such as laboratory mammals with *ad libitum* access to rodent chow (Bellinger et al. 2006) or even humans in modern food-rich societies (Ravelli et al. 1976). Considering how behavioral responses to early life stress interact with ecological context (e.g., the relative ability to acquire food later in life) may yield significant insights into the health or fitness consequences of stress-induced carryover effects.

Our findings that larval corticosterone exposure can have carryover effects on juvenile behaviors support the idea that post-metamorphic life is not a “new beginning” but can be influenced by larval experience (Pechenik 2006). While the fitness ramifications of these corticosterone-induced carryover effects for *S. bombifrons* juveniles remain unclear, it is possible that these effects could be detrimental. As juvenile *S. bombifrons* must prioritize growth to avoid desiccation or predation prior to sexual maturity (Newman and Dunham 1994), it is possible that carryover effects such as increased locomotor activity could impose energetic costs that might reduce juvenile growth or energy reserves. While it is possible that such energetic costs could be offset if increased food-seeking behavior of corticosterone-treated individuals translates to greater caloric intake, future studies are required to understand the fitness or performance consequences of such corticosterone-induced carryover effects under more natural conditions.

Although the larval corticosterone treatment significantly affected multiple juvenile behaviors (Table 1), we found evidence of significant repeatability only for one behavioral trait: latency under control (EtOH-treated) conditions (Table S2). As the behavioral trials were conducted ∼6 weeks apart, it is possible that differences in juvenile age or habituation to the behavioral arena between trials contribute to the lack of observed repeatability (Bell et al. 2009), but it is not possible to disentangle these factors. While evidence for individual repeatability of behavior is mixed (Kelleher et al. 2018), some work suggests that behavior can become more repeatable under stressful conditions, such as predation risk (Toscano et al. 2014; Ehlman et al. 2019). Future studies might consider whether diet type–induced carryover effects on juvenile behavior are more repeatable under conditions that might be more stressful than those imposed in this study, such as nutrient restriction, predation cues, or pond drying scenarios.

Despite carryover effects of larval corticosterone exposure on juvenile behaviors (Figs. 2 and 3) and evidence that the larval corticosterone treatment was sufficient to elevate corticosterone levels at the larval stage (Fig. 1B), we found no effect of larval corticosterone exposure on juvenile corticosterone levels (Table 2). We did find, however, that following stress induction at the juvenile stage, individuals displayed significantly higher corticosterone levels than under pre-stress (i.e., baseline) conditions (Fig. 4), indicating that juveniles were reactive to stress regardless of larval corticosterone treatment. Overall, these findings do not mirror previous observations in *S. bombifrons* that the larval shrimp diet increases juvenile corticosterone levels (Fig. 5; Ledón-Rettig and Lagon 2021; Ledón-Rettig et al. 2023). Our results are also inconsistent with a previous study by Hu et al. (2008) showing that larval corticosterone exposure does influence post-metamorphic corticosterone levels in *Xenopus laevis*, but we note that this study applied a lower corticosterone dose for a longer exposure duration during later stages of tadpole development. We cannot rule out the possibility that larval corticosterone exposure might affect post-metamorphic stress reactivity in a dose-dependent manner or during particular developmental windows. Also, since our analysis was limited to a single clutch, we cannot rule out the possibility that there is family-level variation for post-metamorphic responses or whether these responses are influenced by maternal effects. It is also possible that the novel shrimp diet influences post-metamorphic phenotypes through mechanisms beyond just corticosterone-mediated reprogramming. Future studies may consider the involvement of other known physiological mechanisms involved in responses to nutrient stressors, such as oxidative stress responses (Sanz et al. 2004; Moatt et al. 2020) or nutrient-sensing regulatory pathways such as insulin signaling (Regan et al. 2020).

In summary, our study shows that glucocorticoids likely play a mechanistic role in behavioral carryover effects induced by a novel diet type, yet these diet-induced carryover effects are not driven by glucocorticoids alone. Future work may expand on this “phenotypic engineering” approach to deepen our understanding of the mechanistic basis of diet type–induced carryover effects by considering the potential involvement of corticosterone-independent mechanisms such as alternate hormonal pathways or the direct influence of nutrition on brain development and behavior. A complete understanding of the evolution and development of carryover effects will ultimately require such an integration of evolutionary and developmental approaches (Moore and Martin 2019).

## Supplementary Material

obae012_Supplemental_Files

## Data Availability

Data are included in the supplementary material.
