# Supplementary material for: Corticosterone Contributes to Diet-Induced Reprogramming of Post-Metamorphic Behavior in Spadefoot Toads
Source: Integr Org Biol. 2024 Apr 24;6(1):obae012. doi: 10.1093/iob/obae012 (PMC11067961; doi:10.1093/iob/obae012)
Supplement: obae012_Supplemental_Files [file obae012_supplemental_files.zip › Shephard_Table_S3.docx]

|  | **Fixed effects** | ***X^2^*** | ***d. f.*** | ***p*** |
| --- | --- | --- | --- | --- |
| **Total prey strikes** | Larval diet treatment | 0.86 | 1 | 0.35 |
|  | Juvenile mass | 1.12 | 1 | 0.29 |
| **Distance travelled** | Larval diet treatment | 1.40 | 1 | 0.23 |
|  | Juvenile mass | 0.070 | 1 | 0.79 |
| **Average speed** | Larval diet treatment | 1.88 | 1 | 0.17 |
|  | Juvenile mass | 0.059 | 1 | 0.80 |
| **Average acceleration** | Larval diet treatment | 4.09 | 1 | 0.043 |
|  | Juvenile mass | 0.17 | 1 | 0.68 |
