# Supplementary material for: Corticosterone Contributes to Diet-Induced Reprogramming of Post-Metamorphic Behavior in Spadefoot Toads
Source: Integr Org Biol. 2024 Apr 24;6(1):obae012. doi: 10.1093/iob/obae012 (PMC11067961; doi:10.1093/iob/obae012)
Supplement: obae012_Supplemental_Files [file obae012_supplemental_files.zip › Shephard_Table_S1.docx]

|  | **Spearman’s rho** | ***p*** |
| --- | --- | --- |
| **Latency to move** | 0.17 | 0.16 |
| **Total prey strikes** | -0.057 | 0.64 |
| **Prey strike efficiency** | 0.0045 | 0.97 |
| **Distance travelled** | -0.12 | 0.33 |
| **Average speed** | -0.096 | 0.44 |
| **Average acceleration** | -0.14 | 0.27 |
| **Exploration rate** | -0.084 | 0.50 |
| **Baseline CORT** | 0.22 | 0.30 |
| **Stress-induced CORT** | -0.090 | 0.72 |
