# Supplementary material for: Corticosterone Contributes to Diet-Induced Reprogramming of Post-Metamorphic Behavior in Spadefoot Toads
Source: Integr Org Biol. 2024 Apr 24;6(1):obae012. doi: 10.1093/iob/obae012 (PMC11067961; doi:10.1093/iob/obae012)
Supplement: obae012_Supplemental_Files [file obae012_supplemental_files.zip › Shephard_Table_S2.docx]

|  | **Larval hormonal treatment** | **Estimate of repeatability** | **95% CI** |
| --- | --- | --- | --- |
| **Latency to move** | EtOH | 0.645 | 0.243, 0.90 |
|  | CORT | 0 | 0, 0.482 |
| **Total prey strikes** | EtOH | 0.018 | 0, 0.573 |
|  | CORT | 0.015 | 0, 0.514 |
| **Prey strike efficiency** | EtOH | 0 | 0 |
|  | CORT | 0 | 0, 0 |
| **Exploration rate** | EtOH | 0.007 | 0, 0.738 |
|  | CORT | 0.173 | 0, 0.606 |
| **Distance travelled** | EtOH | 0 | 0, 0 |
|  | CORT | 0 | 0, 0.494 |
| **Average speed** | EtOH | 0 | 0, 0 |
|  | CORT | 0 | 0 0.483 |
| **Average acceleration** | EtOH | 0.075 | 0, 0.721 |
|  | CORT | 0 | 0, 0.142 |
