# Supplementary figures and images for: Corticosterone Contributes to Diet-Induced Reprogramming of Post-Metamorphic Behavior in Spadefoot Toads
Source: Integr Org Biol. 2024 Apr 24;6(1):obae012. doi: 10.1093/iob/obae012 (PMC11067961; doi:10.1093/iob/obae012)

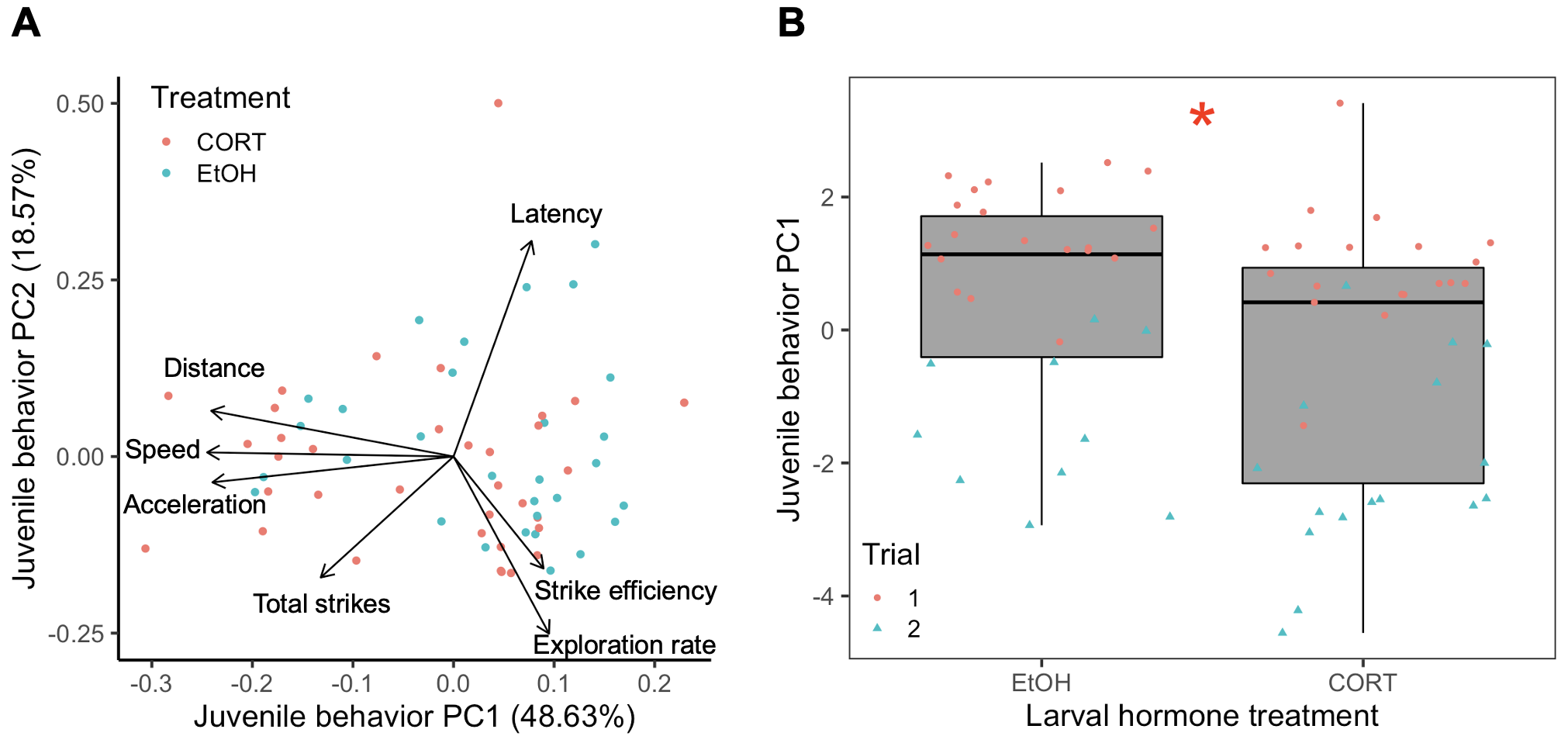

Supplement: obae012_Supplemental_Files [file obae012_supplemental_files.zip › Shephard_Figure_S1_TIFF.tiff]

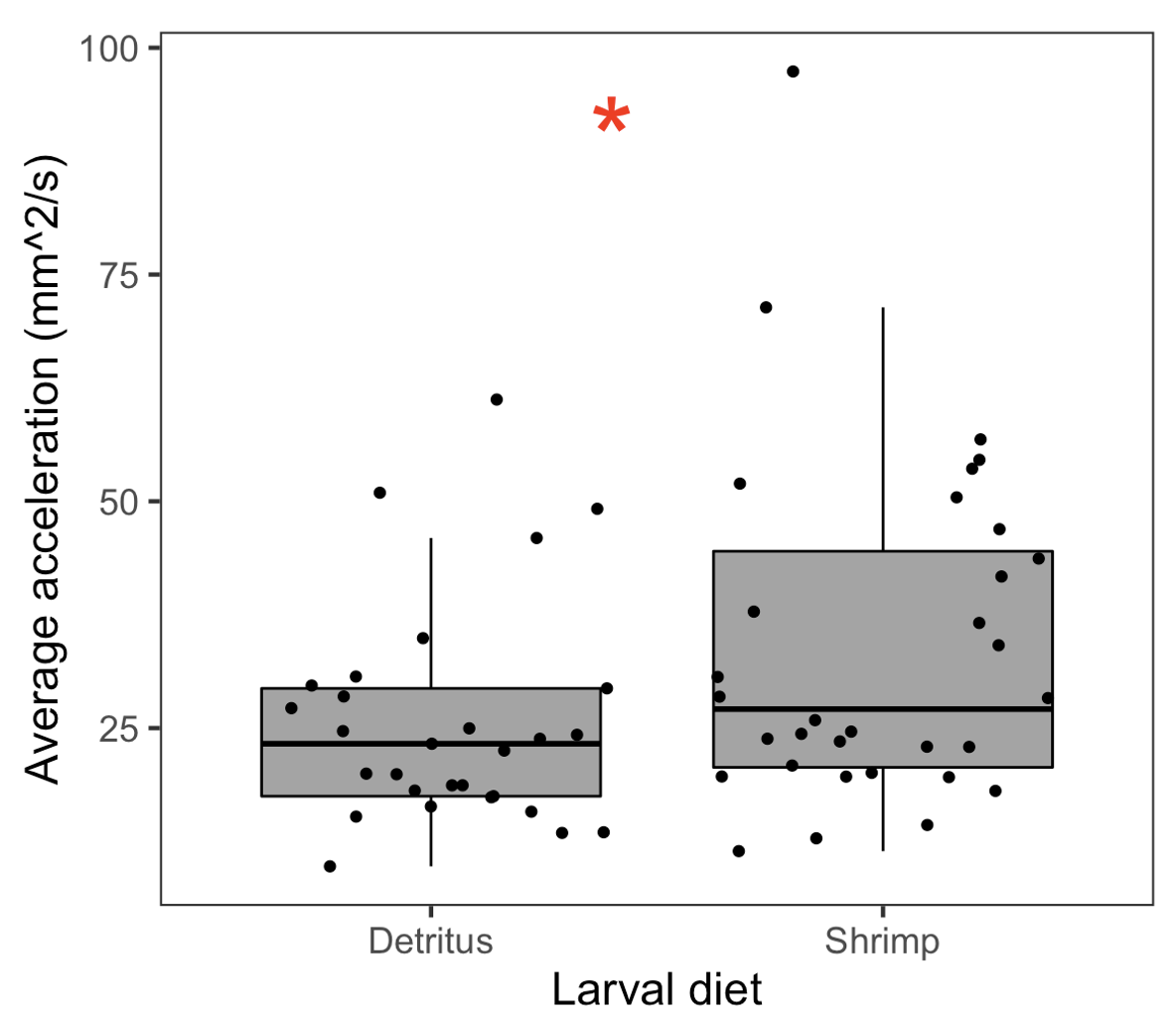

Supplement: obae012_Supplemental_Files [file obae012_supplemental_files.zip › Shephard_Figure_S2_TIFF.tiff]

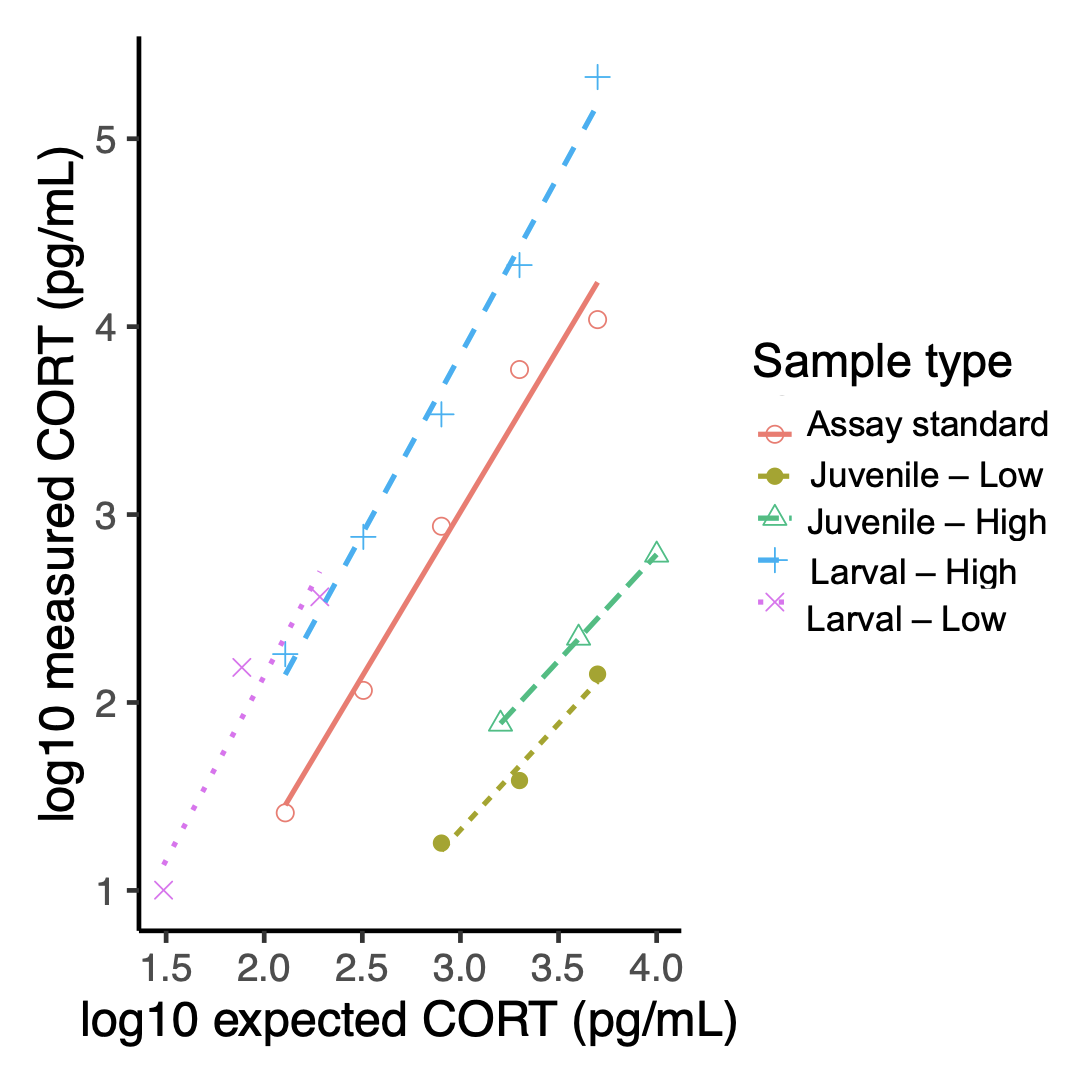

Supplement: obae012_Supplemental_Files [file obae012_supplemental_files.zip › Shephard_Figure_S3_TIFF.tiff]
